# Characterization of Antimicrobial Peptides toward the Development of Novel Antibiotics

**DOI:** 10.3390/ph6081055

**Published:** 2013-08-21

**Authors:** Wataru Aoki, Mitsuyoshi Ueda

**Affiliations:** 1 Japan Society for Promotion of Science, Sakyo-ku, Kyoto 606-8502, Japan; E-Mail: w-aoki@ap.eng.osaka-u.ac.jp; 2 Division of Applied Life Sciences, Graduate School of Agriculture, Kyoto University, Sakyo-ku, Kyoto 606-8502, Japan; 3 Department of Applied Physics, Division of Precision Science & Applied Physics, Graduate School of Engineering, Osaka University, Suita, Osaka 565-0871, Japan

**Keywords:** antibiotic, antimicrobial peptide, drug delivery, activity regulation

## Abstract

Antimicrobial agents have eradicated many infectious diseases and significantly improved our living environment. However, abuse of antimicrobial agents has accelerated the emergence of multidrug-resistant microorganisms, and there is an urgent need for novel antibiotics. Antimicrobial peptides (AMPs) have attracted attention as a novel class of antimicrobial agents because AMPs efficiently kill a wide range of species, including bacteria, fungi, and viruses, via a novel mechanism of action. In addition, they are effective against pathogens that are resistant to almost all conventional antibiotics. AMPs have promising properties; they directly disrupt the functions of cellular membranes and nucleic acids, and the rate of appearance of AMP-resistant strains is very low. However, as pharmaceuticals, AMPs exhibit unfavorable properties, such as instability, hemolytic activity, high cost of production, salt sensitivity, and a broad spectrum of activity. Therefore, it is vital to improve these properties to develop novel AMP treatments. Here, we have reviewed the basic biochemical properties of AMPs and the recent strategies used to modulate these properties of AMPs to enhance their safety.

## 1. Introduction

Since the discovery of the first antibiotic, penicillin [1], public health has been significantly improved by the subsequent development of a variety of antibiotics. The discovery of streptomycin, the first antimicrobial agent effective against tuberculosis, is one of the greatest milestones in medical history [2]. Antibiotics have significantly improved the living environment of human beings, and have cured previously incurable infections. Currently, the leading causes of death in developed countries are non-infectious diseases such as cancer, heart diseases, and cerebrovascular diseases [3]. The development of various antibiotics has inspired the concept of “disease eradication” in the human world; however, overuse of antibiotics has caused the emergence of multidrug-resistant microorganisms. Indeed, strains of important human pathogens, such as *Mycobacterium tuberculosis* [4,5], *Pseudomonas aeruginosa* [6], *Staphylococcus aureus* [7], and *Acinetobacter baumannii* [8], now exhibit increased resistance to almost all conventional antibiotics and the elimination of these adapted strains has become increasingly difficult. To overcome this problem, the synthetic antibiotic linezolid, which belongs to a novel oxazolidinone class of antibiotics, was developed and has been found to be effective against multidrug-resistant *M. tuberculosis* [9] and *S. aureus* [10]. Linezolid binds to the 23S subunit of the ribosome and prevents the formation of the initiation complex [11]. It was hoped that linezolid would be a panacea for infections caused by antibiotic-resistant strains; however, an outbreak of linezolid-resistant *S. aureus* has already been reported [12]. Drug-resistant microorganisms have become a global concern, leading to ceaseless demands for novel antibiotics.

Antimicrobial peptides (AMPs) have received much attention as a novel class of antibiotics. AMPs are peptide antibiotics characterized by an amphipathic nature derived from their positive charges and hydrophobic amino acid residues [13,14]. Since the isolation of the first AMPs, the magainins, from the skin of the African clawed frog *Xenopus laevis* by Zasloff *et al.* [15,16,17], AMPs have been shown to function as an essential component of innate immunity against pathogenic organisms and have evolved in most living organisms over 2.6 billion years [18,19]. AMPs exhibit surprisingly diverse mechanisms of action that are different from those of conventional antibiotics. AMPs disrupt membrane structure, inhibit protein and DNA synthesis, and repress cellular processes, including protein folding, cell wall synthesis, and metabolic turnover [20,21]. Due to these diverse mechanisms of action, AMPs have strong antimicrobial activity in the nanomolar or micromolar range against a broad spectrum of microorganisms, including Gram-positive and Gram-negative bacteria, fungi, and viruses [22,23]. In addition, they are also effective against pathogenic organisms that are resistant to conventional drugs [24]. Therefore, AMPs have been considered as potential future antibiotics.

Despite their great potentials, AMPs have several drawbacks that severely limit their clinical utility, including hemolytic activity [25], broad spectrum of activity [26], rapid turnover in the human body [27], deactivation by high salt concentrations [28], and high cost of production [13]. For example, AMPs can directly interact with host cells and lyse them [29,30]. Furthermore, their broad spectrum of activity can also cause severe problems. Administration of broad-spectrum antibiotics can disrupt the indigenous microflora that provides protective colonization against pathogenic organisms, thereby increasing the risks of diarrhea and other fatal infections [31]. Improvement of the above drawbacks will be necessary for clinical application of AMPs. This article reviews the basic properties of AMPs and the progress toward their clinical application. The peptides reviewed in this article are listed in Table 1.

**Table 1:** Antimicrobial peptides reviewed in this article.

Antimicrobial peptide	Sequence	Origin	Description
Magainin 2	GIGKFLHSAKKFGKAFVGEIMNS	*X.* *laevis*	First AMP isolated from *X. laevis*
Lactoferricin	GRRRRSVQWCA	*Homo* *sapiens*	AMP derived from lactoferrin
Buforin II	TRSSRAGLQFPVGRVHRLLRK	*Bufo* *gargarizans*	AMP derived from histone H2A
Drosocin	GKPRPYSPRPTSHPRPIRV	*Drosophila* *melanogaster*	The Thr residue is *O*-glycosylated.
Pyrrhocoricin	VDKGSYLPRPTPPRPIYNRN	*Pyrrhocoris* *apterus*	Inducible AMP of a sap-sucking insect
Apidaecin	GNNRPVYIPQPRPPHPRL	*Apis* *mellifera*	Isolated from the lymph fluid of honeybees
Lasioglossin-III	VNWKKILGKIIKVVK	*Lasioglossum* *laticeps*	AMP derived from bee venom
HNP1	ACYCRIPACIAGERRYGTCIYQGRLWAFCC	Neutrophils	Human defensins stored in azurophil granules
HNP2	CYCRIPACIAGERRYGTCIYQGRLWAFCC	Neutrophils	
HNP3	DCYCRIPACIAGERRYGTCIYQGRLWAFCC	Neutrophils	
HNP4	VCSCRLVFCRRTELRVGNCLIGGVSFTYCCTRV	Neutrophils	
HBD1	DHYNCVSSGGQCLYSACPIFTKIQGTCYRGKAKCCK	Epithelial cells	Human defensins secreted by epithelial cells
HBD2	TCLKSGAICHPVFCPRRYKQIGTCGLPGTKCCKKP	Epithelial cells	
HBD3	GIINTLQKYYCRVRGGRCAVLSCLPKEEQIGKCSTRGRKCCRRKK	Epithelial cells	
HBD4	ELDRICGYGTARCRKKCRSQEYRIGRCPNTYACCLRK	Epithelial cells	
RTD1	GFCRCLCRRGVCRCICTR	Primate	Premature stop codons in the human θ-defensin sequence
Melittin	GIGAVLKVLTTGLPALISWIKRKRQQ	*A.* *mellifera*	Peptide antibiotic with toxicity to human cells
Gramicidin S	VOrnLdFPVOrnLdFP	*Bacillus* *brevis*	Peptide antibiotic with toxicity to human cells
Adepantin 1	GIGKHVGKALKGLKGLLKGLGES	Artificial	Predicted by AMPad to have low hemolytic activity
R5L	PLCRCRVRPYRCRCVG	Artificial	Designed to mimic the LPS-binding sites of LBP, cyclic
Oncocin	VDKPPYLPRPRPPRRIYNR	Artificial	Proline-rich, Gram-selective AMP
M8G2	TFFRLFNRGGGKNLRIIRKGIHIIKKY	Artificial	Designed using STAMP technology to target *Streptococcus mutans*
Clavanin A	VFQFLGKIIHHVGNFVHGFSHVF	Styela clava	Histidine-rich, pH-dependent AMP
AAP2	FHFFHHFFHFFHHF	Artificial	Acid-activated AMP based on clavanin A
Protease-activated AMP	DDAEAVGPEAFADEDLDEGFIKAFPKRRWQWRMKKLG	Artificial	Protease-activated AMP based on lactoferricin

AMP, antimicrobial peptide; Orn, ornithine; dF, d-isoform of phenylalanine; Thr, threonine; LPS, lipopolysaccharide; LBP, LPS-binding protein; STAMP, specifically targeted antimicrobial peptide.

## 2. Mechanism of Action of AMPs

Cationic AMPs are amphipathic peptides characterized by a significant proportion of hydrophobic amino acid residues and an overall positive charge. In this section, we have reviewed the mechanism underlying the action of AMPs by using the example of lactoferricin, which is one of the most extensively studied AMPs [32]. Lactoferricin (GRRRRSVQWCA) is naturally produced through proteolysis of lactoferrin by pepsin under acidic conditions [33]. Lactoferricin is rich in arginine and hydrophobic valine and tryptophan residues and possesses strong antimicrobial activity against multidrug-resistant pathogens, including *S. aureus* [34], *A. baumannii* [35], and *Candida albicans* [36,37].

**Figure 1:**
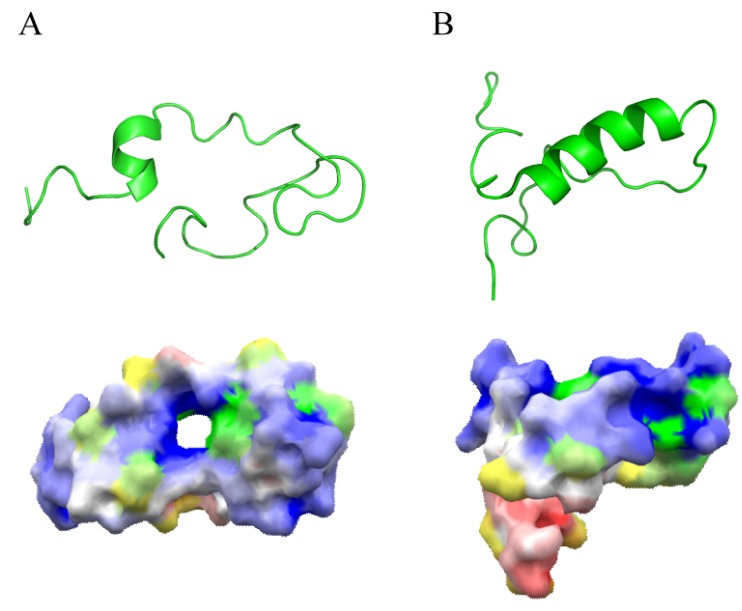
Structure of lactoferricin. (**A**) Representative structure and electrostatic surface display of lactoferricin in aqueous solvent (PDB accession, 1Z6W). (**B**) Representative structure and electrostatic surface display of lactoferricin with a distinct α-helix in membrane-mimetic solvent (PDB accession, 1Z6V). The negatively charged surface is shown in red, and the positively charged surface in blue. The molecular surface corresponding to the hydrophobic residues was also indicated by addition of yellow color to the electrostatic potential color; hence, orange color indicates hydrophobic and negatively charged surface; yellow color shows hydrophobic and neutral surface; green color indicates hydrophobic and positively charged surface.

Lactoferricin and other linear AMPs disrupt bacterial cell membranes by drastically changing their tertiary structure depending on the surrounding environment [38,39]. Nuclear magnetic resonance spectroscopy has shown that in aqueous solution, AMPs adapt a partially folded structure (Figure 1A) [40,41]. By contrast, the membrane-mimetic environment in detergents induces a significantly amphipathic helix structure that separates all the hydrophobic residues to one side, and the positively charged residues to the other (Figure 1B) [33,41,42].

**Figure 2:**
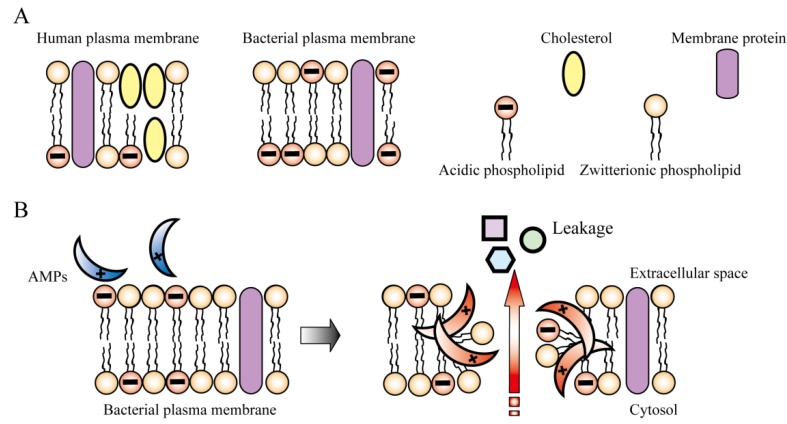
Mechanism of action of antimicrobial peptides (AMPs). (**A**) Comparison of human and bacterial plasma membranes. (**B**) Disruption of bacterial membrane by AMPs. AMPs preferentially interact with bacterial plasma membrane due to their electrical charge. When AMPs interact with the negatively charged bacterial plasma membrane, they spontaneously form pores and disrupt membrane integrity.

AMPs with positive charges are electrostatically attracted to the negatively charged surface of the bacterial cytoplasmic membrane. Subsequent to their interaction with the bacterial membrane, AMPs spontaneously oligomerize and form transmembrane pores that cause leakage of the cellular contents (Figure 2) [20]. Indeed, replacement of positively charged arginine or hydrophobic tryptophan residues with alanine causes significant reduction in antibacterial activity. This result suggests importance of positively charged amino acids and hydrophobic amino acids to interact with bacterial membrane and to form transmembrane pores, supporting the above mechanism of action [43].

Although AMPs possess antimicrobial activity that disrupts bacterial membrane integrity, other modes of action targeting key cellular processes, including DNA and protein synthesis, protein folding, cell wall synthesis, and metabolic turnover, have been characterized [20,44]. Thus, transmembrane pore formation is not the only mechanism, making it necessary to carefully determine how each AMP kills microorganisms. For example, buforin II (Table 1), an AMP isolated from the stomach tissue of the Asian toad *Bufo gargarizans*, penetrates the cell membrane and strongly inhibits the functions of DNA and RNA in cells [45,46]. Drosocin, pyrrhocoricin, and apidaecin, originally isolated from insects, act on heat shock proteins (DnaK and GroEL) and repress the stress response of cells [47]. Recent studies have revealed novel alternative functions for AMPs, including neutralization of endotoxins [48], wound healing [49], cytotoxicity against neoplastic cells [50], and immunomodulation [51]. Some peptides can even use multiple antimicrobial mechanisms [44]. This multiple-hit strategy may be effective in increasing antimicrobial efficiency and evading potential resistance mechanisms.

The acquisition of resistance to AMPs is very rare, compared to conventional antibiotics, because microorganisms are killed by direct disruption of cellular components, including the microbial membrane and DNA [16,52]. The appearance of AMP-resistant strains is less likely because development of microbial resistance by gene mutation to such a microbicidal mechanism of action is difficult [53], although microorganisms can coordinate countermeasures to circumvent the attack by AMPs to some extent [54].

For example, *S. aureus*, a Gram-positive bacterium, exhibits a higher minimum inhibitory concentration (MIC) for AMPs by modifying its cell surface teichoic acid with d-alanine [55,56]. Though teichoic acids are polyanionic, incorporation of d-alanine reduces the negative charges on the cell wall. Furthermore, *S. aureus* can modify its membrane lipid phosphatidylglycerol via enzymatic transfer of a lysine [57]. These modifications weaken the interaction between cationic AMPs and the cell wall, leading to acquisition of resistance. *P. aeruginosa* produces elastase, a serine protease that hydrolyzes amides and esters, in a wound fluid environment and degrades the AMP LL-37 [58]. The production of bacterial elastase degrades AMPs and enhances the survival of *P. aeruginosa*. Another resistance mechanism is external trapping of AMPs through the actions of bacterial surface-associated or secreted proteins. These interfering molecules neutralize AMPs and reduce bacterial killing. *S. aureus* produces staphylokinase, which directly binds to defensins produced by host immune cells [59]. Staphylokinase neutralizes AMPs, and the formation of the kinase-AMP complex results in complete inhibition of bactericidal effects.

Recent studies have reported an interesting resistance mechanism of a dysentery bacillus. *Shigella flexneri*, which causes dysentery, lowers host innate immunity by targeting the expression of host defensins [60]. Upon infection, *S. flexneri* produces MxiE, a transcriptional activator, which modulates the expression of host defensins. Another study reported the possibility that *S. flexneri* might use host defense molecules to enhance virulence and subvert innate immunity [61]. Neutrophils are the first line of defense and an essential component of innate immunity; they kill pathogenic microorganisms via phagocytosis, neutrophil extracellular traps [62], and degranulation [63]. During the process of degranulation, the *Shigella* cell surface binds to cationic granular antimicrobial proteins, causing increased adhesion and hyperinvasion [61]. This effect is considered to be caused by surface negative charges because a lipopolysaccharide (LPS) mutant has been found to show enhanced hyperinvasion.

## 3. Improvement of AMPs for Clinical Use

Despite many attractive properties, AMPs possess several disadvantages that impede their development as therapeutic agents. These disadvantages include hemolytic activity toward human cells, rapid turnover in the human body, reduced activity based on salt sensitivity, and high cost of production. Given these attributes, the challenges in developing AMP-based pharmaceuticals remain formidable; however, optimization of peptide sequences or chemical modification of AMPs could overcome these barriers and facilitate their development for commercial use. In this section, we have reviewed strategies to solve these problems.

### 3.1. Hemolytic Activity

Although AMPs bind to bacterial surfaces via electrostatic interactions, some types of AMPs can directly interact with host cells and lyse them [25]. The ratio of antimicrobial activity to hemolytic activity is defined as the therapeutic index, and a high therapeutic index is necessary for avoiding hemolysis of host cells [64]. To solve the issue of hemolysis, it is important to use non-hemolytic AMPs as seed compounds.

It would also be beneficial to optimize the peptide sequences to decrease hemolytic activity. Many naturally occurring AMPs are amidated at the *C* terminus; amidated peptides exhibit higher antimicrobial activity but are also more hemolytic than that of natural AMPs [65]. Ulrich *et al*. reported that *C*-terminal deamidation of AMPs reduces undesired hemolytic activity while maintaining antimicrobial effects [66]. It is known that *C*-terminal amidation stabilizes amphipathic helix formation of AMPs upon their binding to the membrane bilayer, leading to strong activity [67,68]. Furthermore, structure-function analysis has revealed that high amphiphilicity and high hydrophobicity contribute to increased hemolysis, whereas an inverse correlation exists between antimicrobial activity and these properties [69,70,71]. Changes in hydrophobic moment and amphiphilicity are likely to be important for minimizing side effects.

### 3.2. Rapid Turnover in the Human Body

AMPs are rapidly degraded in the human body by proteases; therefore, proteolytic stability is essential for therapeutic use. Protease susceptibility can be overcome using various strategies, including d-isomerization, incorporation of chemical compounds, cyclization, and use of peptide mimetics. This section reviews the effects of the above strategies.

In general, the d-isomer chiral counterparts of l-isomer AMPs show greater antimicrobial activity than their l-isomer AMPs because host and bacterial proteases cannot hydrolyze the unnatural d-isomers [72,73]. This observation is consistent with the hypothesis that antimicrobial activity is mediated by a chirality-independent interaction with membrane and nucleic acids [74]. Malmsten *et al*. evaluated four strategies (tryptophan substitution, terminal amidation, acetylation, and d-isomerization) for improving the proteolytic resistance of AMPs [75]. The authors found that d-isomerization was the best strategy to protect AMPs; the other three methods also improved proteolytic stability to some extent. The authors also found that end-modification resulted in higher antimicrobial potency than that of d-isomerized AMPs. Although d-isomerization confers good stability, the strategy is not cost-effective and is not applicable to chirality-dependent AMPs.

Vogel *et al*. investigated the effects of end-capping and cyclization on the stability of AMPs [76]. End-capping was found to exert contradictory effects on antimicrobial activity and proteolytic stability. *C*-terminal amidation had little effect on protease resistance in human serum, but increased peptide activity. On the other hand, *N*-terminal acetylation significantly increased proteolytic stability, but decreased peptide activity. Peptide cyclization was highly effective for both serum stability and microbicidal activity, suggesting that cyclization is a good approach for improving the pharmacodynamics of AMPs.

Incorporation of chemical compounds could improve the stability of AMPs. Kumar *et al*. reported that fluorinated derivatives of buforin and magainin displayed ~2.3-fold higher proteolytic stability while retaining or exhibiting higher antimicrobial activity [77]. While further investigation is necessary, the effect could be attributed to the increased steric bulk of the fluorinated residues or to electronic perturbation of the amide bond [77]. It is likely that steric occlusion of the peptides from the active site of proteases leads to increased proteolytic stability.

**Figure 3:**
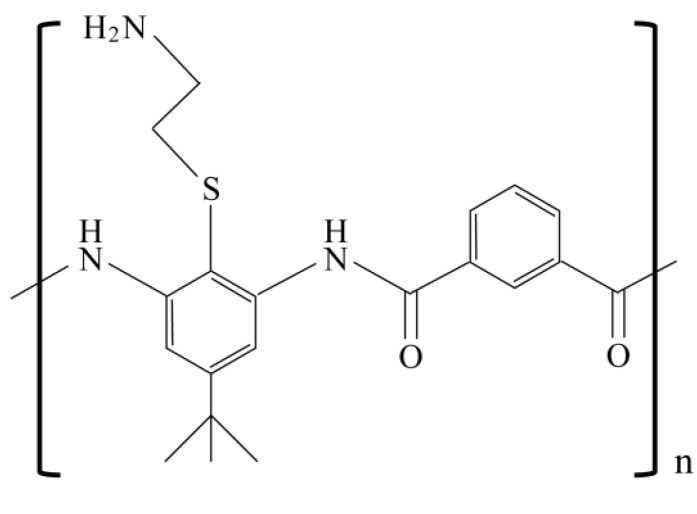
Representative structure of arylamide polymers. The diamine was used for convenience of synthesis and its conformational properties. The thioether was chosen with the expectation that hydrogen bonding to both the amide protons would stiffen the conformation.

The design of polymers that mimic the complex structures of AMPs is an important endeavor with practical implications [78]. De Grado *et al*. designed a series of amphiphilic arylamide polymers (peptide mimetics) that captured the physical properties of AMPs [79] (Figure 3). The arylamide derivatives showed bactericidal activity against *Escherichia coli* and *P. aeruginosa* at an MIC level (μg/mL) comparable to that of conventional antibiotics. The mechanism of action of the polymers is similar to that of AMPs, which disrupt bacterial cell membranes [80]. The polymers do not have peptide bonds, and they exhibit high stability against proteases. It is also noteworthy that arylamide polymers are easy to prepare from inexpensive monomers. Recently, the authors succeeded in improving the pharmacokinetics of arylamide polymers in animal models [81]. The authors synthesized very small arylamide polymers (600–1,000 Da). The polymers were rigidified via hydrogen bond formation and were highly active against *S. aureus* and *E. coli* in an animal model. The *in vivo* efficacy may be related to their stability, tissue distribution, and pharmacokinetics, which have not yet been fully investigated.

### 3.3. Reduced Activity due to Salt Sensitivity

AMPs need electrostatic interaction with microbial membranes to form secondary structures. This step is salt sensitive and often causes problems in clinical application [82]. Human body fluids containing high salt concentrations deactivate many AMPs; therefore, it is necessary to develop salt-insensitive AMPs. Stabilization of the secondary structure of AMPs could confer salt insensitivity. Kim *et al*. used helix-capping motifs to stabilize the peptide structures of helical AMPs [83]. The peptide sequences, APKAM for the *N*-terminus and LQKKGI for the *C*-terminus, were incorporated into helical AMPs as a helix stabilizer. While many natural AMPs lose their activity in 200 mM NaCl, peptides with helix-capping motifs retained significantly stronger activity upon exposure to the same concentration of NaCl. Furthermore, the peptides showed high activity in an animal infection model of *Streptococcus pyogenes* and the host tissues remained intact, indicating antimicrobial activity at physiological salt concentrations with minimal side effects.

Cheng *et al*. developed an easy strategy to increase salt resistance by replacing the tryptophan or histidine residues of AMPs with the bulky β-naphthylalanine and β-(4,4'-biphenyl)alanine [84]. The peptides displayed antimicrobial activity against multidrug resistant strains at high salt concentrations (150 mM). This is likely due to stabilization of the AMP structure. Moreover, the authors found that end-capping of AMPs via β-naphthylalanine addition further boosted salt resistance and serum stability, whereas the antimicrobial activities of the modified peptides were less affected [85]. An example of naturally-occurring salt-resistant AMP is lasioglossin-III (Table 1), found in *Lasioglossum laticeps* [86]. Lasioglossin-III showed strong antimicrobial effect against *E. coli*, *S. aureus*, and *P aeruginosa* even under physiological salt concentration (150 mM NaCl and 2 mM MgCl_2_) while the resistant-mechanism is still unclear. Finding of the salt-resistant AMP will motivate us to screen useful AMPs from nature.

### 3.4. High Cost of Production

Though large quantities of AMPs are required for clinical trials, the cost of production of AMPs is very high compared to that of conventional antibiotics [87]. Therefore, a suitable production method is essential to develop AMPs as novel pharmaceuticals. Furthermore, heterologous production of AMPs in prokaryotic systems tends to be difficult because AMPs are toxic to prokaryotic cells. In this section, we have reviewed several strategies to reduce the toxicity for host cells and obtain large amounts of AMPs.

Fusion expression using solubility-enhancing carriers is the most popular strategy to obtain a high yield of AMPs. The representative carrier is thioredoxin, which is characterized by its small size (11.8 kDa) and high solubility [88]. Recently, small ubiquitin-related modifier (SUMO) has been shown to be a good carrier for improving the solubility of target proteins and peptides [89]. This SUMO technology is also applicable to AMPs. Kalman *et al*. achieved large-scale industrial production of AMPs in bacteria by using the SUMO technology [90]. The authors constructed an expression vector encoding fusion peptides of AMPs and SUMO and found that the peptide produced did not exhibit significant toxicity. A yield of 0.08 g/L of pure AMPs has been successfully achieved in pilot-scale fermentation (10 L).

Another production strategy is the use of aggregation-promoting carriers [91]. The production of insoluble AMPs could mask their toxic activity and protect them from degradation by host proteases. Furthermore, expression of insoluble AMPs enables their quick purification using a simple centrifugation procedure. Hong *et al*. used fusion peptides of AMPs and a truncated *E. coli* PurF fragment that has a high tendency to form inclusion bodies [92,93]. Several AMPs have been successfully produced using this system, and high yields have been achieved using SP-Sepharose cation-exchange chromatography.

Acidic peptide-mediated production of AMPs could inhibit toxic activity in heterologous expression systems. The cationic charges of AMPs are required for their activity; neutralization of the charges by fusion with an anionic peptide leads to deactivation of AMPs. Kim *et al*. constructed a fusion peptide of buforin II and an acidic peptide called magainin intervening sequence [94]. Magainin intervening sequence is rich in acidic amino acids and has been used in nature to inhibit the toxic effects of magainin in *X. laevis* [15]. Using this sequence, the authors achieved an AMP yield of 107 mg/L of *E. coli* culture. This technique has universal applicability and has been used in the production of other AMPs [95].

## 4. Temporal and Spacial Regulation of AMPs in Nature

AMPs have several disadvantages and their activity is limited under physiological conditions; however, the human body efficiently uses AMPs as innate immunity, a front line defense against pathogenic organisms. It is evident that AMPs play important roles in protecting against fatal diseases, given that many types of diseases are caused by gene mutations that inactivate AMPs [96]. Investigation of the mechanisms of action *in vivo* will provide valuable information to develop effective AMPs for clinical use. In this section, we have reviewed our current understanding of the temporal and spatial regulation of AMPs. We have focused on the mechanisms used by multicellular organisms to avoid adverse effects and to effectively kill pathogenic organisms using AMPs.

The human body has a symbiotic relationship with commensal microflora (microbiota), an integral part of complex mucosal surfaces. The human distal gut microbiota is composed of 10^13^–10^14^ microorganism cells, including 72 bacterial phylotypes and one archaeal phylotype [97]. The microbiota exhibits various traits (e.g., methanogenesis) that humans have not been required to evolve on their own [98,99]. Thus, humans are recognized as super-organisms whose metabolic processes represent a mixture of microbial and human attributes. This situation leads to the question of how human bodies can rapidly eliminate pathogenic organisms while maintaining the commensal microbiota.

The innate immune system, including AMPs and pattern recognition receptors such as toll-like receptors (TLRs), plays an important role in determining the microbial population balance of mucosal surfaces. The importance of innate immunity is demonstrated by the existence of severe diseases such as cystic fibrosis [100], Kostmann syndrome [101], and Papillon-Lefèvre syndrome [102], which are caused by local defects in AMP activities. For example, cystic fibrosis can be lethal because of progressive destruction of the airways by recurrent infections and inflammation caused by *P. aeruginosa* [103]. Cystic fibrosis is an autosomal recessive genetic disorder caused by mutations in the cystic fibrosis transmembrane conductance regulator (CFTR). Mutations in CFTR lead to aberrant high salt concentrations in body fluids and deactivation of AMPs, resulting in fatal infections [102,104]. These diseases prove the essential roles of AMPs in normal immune responses.

The oral cavity and the airways are major barriers to pathogenic organisms because most external microorganisms enter the human body via these surfaces [105]. Epithelial cells, neutrophils, and salivary glands in the oral cavity secrete over 45 types of AMPs and antimicrobial proteins; however, the concentrations of these antimicrobial agents do not exceed the MIC for most microbes [106]. Thus, basal AMPs in the oral cavity can be regarded as modulators that maintain the microbiota and prevent outgrowth, rather than weapons that eliminate individual microorganisms. The oral epithelium specifically recognizes a subset of bacteria and TLR ligands and produces stronger AMPs to maintain the balance between health and disease [102].

Defensins, the most studied AMPs in vertebrates, are abundant in nature. The defensin family is evolutionarily conserved in vertebrates, and its members are characterized by the presence of similar structures of β-sheet-rich folds and three disulfide bonds [107]. Primates have three defensin subfamilies, namely, α-defensins, β-defensins, and θ-defensins (retrocyclins such as rhesus theta-defensin, RTD1 shown in Table 1). Each family has different secondary structures. Among them, α-defensins and β-defensins are the two main defensin subfamilies in humans because the human θ-defensin gene has premature stop codons in its genome sequence and has been recognized as a pseudogene [108,109]. In this section, we use the defensin family as an example to describe how human tissues temporally and spatially regulate the activity of AMPs.

### 4.1. Neutrophils

**Figure 4:**
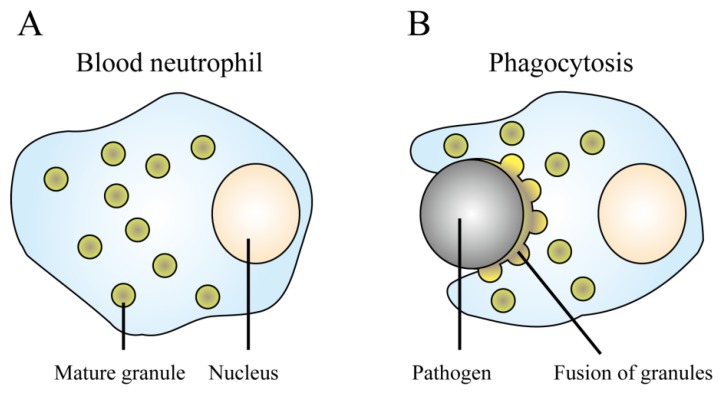
Phagocytosis of pathogenic cells by neutrophils. (**A**) Synthesis of defensins in neutrophils. α-Defensins are proteolytically processed to the mature form and then packaged into azurophil granules. (**B**) Phagocytosis of pathogenic cells. When a mature neutrophil ingests a pathogenic cell, it simultaneously evokes the fusion of azurophil granules and the phagosome, and the α-defensins then exert antimicrobial activity in the limited space.

α-Defensins (human neutrophil peptides, HNP1-4 shown in Table 1) are predominant in neutrophils [110,111], a type of white blood cell (leukocyte), and account for 30%–50% of the total protein content of the azurophil granule, which is an arsenal-storage organelle in leukocytes [112]. Synthesis of α-defensins occurs in the bone marrow where promyelocytes reside [113,114], and the translated α-defensin precursors are proteolytically processed to mature α-defensins and packaged into azurophil granules [115] (Figure 4A). The concentration of intravacuolar α-defensins reaches several grams per liter, which exceeds the MICs for most bacteria [116,117]. When mature neutrophils encounter and ingest pathogenic organisms in phagocytic vacuoles, the azurophil granules fuse with the phagosome and release α-defensins [118,119] (Figure 4B). Because phagosomes have little space, neutrophils are able to attack the pathogens by using the minimally diluted AMPs at a high concentration. Neutrophils use AMPs against phagocytosed organisms in the limited space, leading to high antimicrobial activity and preservation of the good microbiota, with minimal adverse effects. This observation suggests that it is important to specifically concentrate AMPs at sites where pathogens cause diseases.

### 4.2. Epithelial Cells

β-Defensins (human beta-defensins, HBD1-4 shown in Table 1) are mainly produced in epithelial cells. Among them, HBD1 is constitutively produced in epithelial tissues, and it exhibits mild antimicrobial activity compared to HBD2 and HBD3. In addition, its concentration in the mucosal fluid is not high and is less than the MIC for most microbes [106,120]. Thus, HBD1 may be recognized by modulators of the human microbiome. HBD2 is constitutively produced in gingival tissues; in addition, HBD2 is an inducible AMP that is upregulated only in inflamed skin [121,122]. Upregulation of HBD2 is caused by pathogenic organisms via activation of a pathway that requires interleukin-1 and nuclear factor (NF)-κB [123,124]. HBD3 and HBD4 are also recognized as inducible defensins; however, they are regulated by NF-κB-independent mechanisms [125,126]. Based on these observations, it is proposed that HBD1 plays a role in the maintenance of steady-state microflora in epithelial tissues, whereas HBD2-4 function as effective antibiotics against pathogens.

## 5. Design of Molecular-Targeted AMPs

Although AMPs are promising antibiotics, their disadvantages hamper their development as effective pharmaceuticals. One representative problem is the hemolytic activity of AMPs. Cationic AMPs preferentially interact with negatively charged bacterial membranes; however, at high concentrations, they damage mammalian cells [30]. Under physiological conditions, the salt concentrations in body fluids and serum components inhibit the antimicrobial activity of AMPs. Therefore, under such conditions, AMPs are required at high concentrations, resulting in adverse effects on the host cells [28]. Thus, it is necessary to develop AMPs that exhibit high specificity toward bacteria, even at high concentrations. The other problem may be attributed to their broad spectrum of antimicrobial activity against viruses, fungi, and Gram-positive and Gram-negative bacteria. Highly concentrated AMPs can destroy almost the entire microbiota. The indigenous microflora provides protective colonization against pathogenic organisms, and therefore, administration of broad-spectrum antibiotics increases the risks of diarrhea and other fatal infections. It is known that classical opportunistic pathogens such as *S. aureus*, *Clostridium difficile*, and *C. albicans* cause infections at vacated niches following antibiotic treatments [127,128,129,130]. Thus, there is an urgent need for novel designer AMPs that would allow temporal and spatial regulation of their antimicrobial activity. In this section, we have reviewed the current status of designer target-selective AMPs and their potential as safe antibiotics.

### 5.1. Bacterium-Selective AMPs

It is necessary for AMPs to have a high therapeutic index, which indicates a high bactericidal ability but low toxicity toward human cells. The selectivity of AMPs depends on the differences between bacterial and mammalian cell membranes, namely, the lipid composition and electrical charge (Figure 2A). Positively charged AMPs are selectively attracted to the negatively charged bacterial cell membrane [131,132] (Figure 2B). Unlike the mammalian cell membrane, the bacterial membrane has a net negative charge owing to the presence of acidic phospholipids in the outer membrane layer. Thus, AMPs preferentially attack bacteria. In addition, abundant cholesterol stabilizes the mammalian cell membrane and reduces susceptibility to AMPs [30]. However, non-selective AMPs, such as melittin and gramicidin S (Table 1), exhibit dual activity against both bacteria and mammalian cells [133,134], and it is possible that selective AMPs could also exert a hemolytic effect on human cells at high concentrations.

Structure-function analyses of AMPs have shown that high hydrophobicity and amphipathicity (hydrophobic moment) correlate with increased hemolytic activity [135]. Based on this observation, Tossi *et al*. computationally designed highly selective AMPs using knowledge-based methods [136]. The authors created a structure-selectivity database (AMPad) of previously known AMPs, and programmed a simulator based on the finding that the cosine of the angle between two sequence moments obtained with different hydrophobicity scales can identify highly selective peptide antibiotics. Using the algorithm, they synthesized a 23-residue glycine-rich peptide, adepantin 1 (Table 1), and found that the peptide had a significantly higher bacterium-human selectivity (high therapeutic index) than any other AMP in the AMPad database [137]. Such a computer-assisted method based on previous observations could be a promising approach for designing novel AMPs with high bacterial selectivity.

Recently, Wang *et al*. developed a novel *ab initio* approach to screen artificial AMPs against methicillin-resistant *Staphylococcus aureus* (MRSA) [138]. The authors constructed a database called the antimicrobial peptide database (APD). The APD includes vast amount of AMPs and the peptides in the APD can be sorted according to key parameters, enabling users to effectively retrieve peptides with defined properties. Using the APD, the authors derived the most probable parameters (e.g., net charge, hydrophobicity, and amino acid composition) by developing a database filtering technology, which enables to define peptide parameters. Based on database screening, the authors succeeded in retrieving all the peptide parameters appropriate for MRSA and designing novel AMPs that showed strong activity against MRSA. Such an *ab initio* approach will be a cost-effective and promising method.

### 5.2. Gram Nature-Selective AMPs

Gram-negative bacteria are one of the major causes of hospital-acquired infections [139]. Multidrug-resistant Gram-negative bacteria such as *A. baumannii*, *P. aeruginosa*, and Enterobacteriaceae have been identified worldwide; multidrug resistance would drastically reduce the number of effective antibiotics [140]. Thus, there is a critical need for the development of innovative antimicrobials with novel mechanisms of action. A possible solution is the use of Gram nature-selective AMPs that can recognize the differences in cell surface components [141]. A specific feature of Gram-negative bacteria is that their outer cell surface contains LPS. To develop Gram-negative–selective antimicrobial agents, Tam *et al*. designed constrained cyclic AMPs that mimicked the LPS-binding sites of the LPS-binding proteins [142]. The authors synthesized artificial peptides with head-to-tail cyclic backbones and two cross-bracing disulfides. These peptides, including a peptide named R5L (Table 1), displayed potent activity against Gram-negative bacteria, with >200-fold selectivity over Gram-positive bacteria. Furthermore, these peptides inhibited LPS-induced gelation in the *Limulus* amoebocyte lysate assay at low concentrations, which indicates a possible application of the peptide as a therapeutic agent for neutralizing endotoxin shock [143]. β-Hairpin AMPs and proline-rich AMPs, such as oncocin (Table 1), have also attracted attention because they predominantly target Gram-negative bacteria [144,145,146]. Bactenecins, which are members of the proline-rich cathelicidin family, are also known to bind LPS and specifically kill Gram-negative bacteria [147]. Further development of Gram-positive–selective AMPs will broaden the applications of AMPs.

### 5.3. STAMP Technology

**Figure 5:**
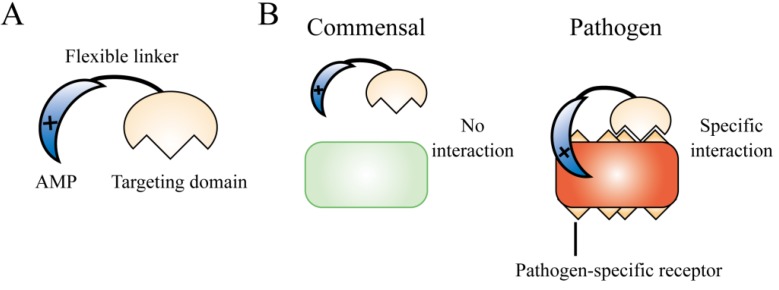
Specifically targeted antimicrobial peptide (STAMP) technology. (**A**) Overview of the STAMP technology. STAMPs are composed of two domains, namely, an AMP domain and a targeting domain. (**B**) A schematic of the selective activity against pathogens. The STAMP accumulates around the targeted pathogenic cells and kills them.

Development of species-selective smart AMPs is an important research area. Shi *et al*. have developed a technology called specifically targeted antimicrobial peptide (STAMP) [148,149]. The STAMP technology is based on the construction of a fusion peptide by combining two functionally independent components, namely, a targeting domain and a killing AMP domain, using a short flexible linker (Figure 5A). The targeting domain confers selectivity on the AMP domain by binding to the pathogen using specific determinants on the pathogen surface, such as membrane hydrophobicity, charge, pheromone receptors, cell wall components, or characteristic virulent attributes. This binding increases the selective accumulation of the AMPs around the pathogenic organisms and significantly increases the local concentration of the peptide (Figure 5B).

*Streptococcus* *mutans* is the most cariogenic of all the oral streptococci [150,151]. Tooth decay is not immediately life threatening, but causes a heavy burden on the economy. Shi *et al*. used the sequence of competence stimulating peptide (CSP), a pheromone produced by *S. mutans*, as the targeting domain [152]. An AMP (M8G2 shown in Table 1) conjugated to CSP via a peptide linker region specifically targeted and killed *S. mutans* cells, but not the other streptococci tested. The peptide was also mixed with the*S. mutans ΔcomD* mutant to determine whether the killing ability depended on a specific interaction between CSP and ComD (a histidine kinase receptor protein), which is the proposed interaction partner of CSP. However, similar antimicrobial activity was observed against the *S. mutans ΔcomD* mutant, indicating the presence of another interaction partner. The STAMP technology could be a promising approach to specifically eliminate pathogenic organisms from the normal microflora. Preservation of good microorganisms could prevent secondary infections and other negative clinical consequences [153].

### 5.4. Environment-Sensing AMPs

Pathogenic organisms often induce dramatic changes in the surrounding host environment because of overgrowth and metabolic activities. The design of novel AMPs that sense environmental changes may lead to the production of a selective antibiotic that exerts antimicrobial activity without perturbing the microbiota. For example, the plaque bacterium *S. mutans* dramatically decreases the oral pH and causes tooth demineralization [154] Therefore, an AMP that is activated by low pH could be an antibiotic specific to acid-generating pathogens, such as *S. mutans*. To test this hypothesis, acid-reactive AMPs were designed based on the pH-dependent AMP clavanin A (Table 1) [155,156]. Clavanin A is rich in histidine residues; therefore, its electrostatic status could dramatically change in response to subtle changes in the surrounding environment. The amino acids of clavanin A were substituted to alter its pH-dependent behavior. As a result, acid-activated peptide 2 (AAP2 shown in Table 1) was constructed with maximum activity at acidic pH 5.5, but no activity at physiological pH 7.5. The antimicrobial activity was highly dependent on the surrounding pH, and correlated with the positive charge of the AAP2 peptide. It is known that C. albicans, a major multidrug-resistant opportunistic fungal pathogen, actively acidifies its surrounding microniches by secreting organic acids [157]. Thus, such a designed peptide could be an alternative pharmaceutical against *C. albicans*.

### 5.5. Protease-Activated AMPs

**Figure 6:**
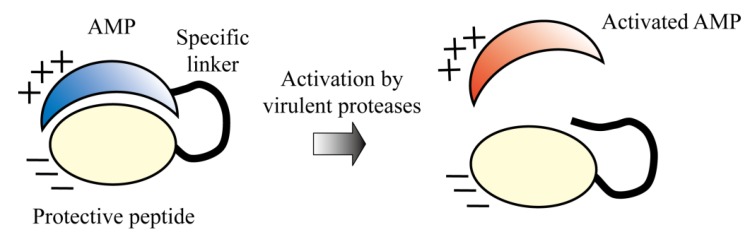
Protease-activated AMP (Table 1). The peptide is composed of three domains: an AMP, a protective peptide, and a specific linker. The antimicrobial activity of the AMP is inhibited by conjunction with the anionic protective peptide. Virulent proteases cleave the specific linker and release the AMP, leading to its activation.

Many pathogenic organisms possess virulent proteases with characteristic substrate specificities. For example, *Plasmodium* spp., the causative organisms of malaria, require proteases for full virulence [158]. *C. albicans* secretes ten types of aspartic proteases to degrade host tissues and evade immune cells [159,160]. Thus, designing an AMP that is activated by virulent proteases may confer high selectivity toward each pathogenic organism. Based on this postulation, Aoki *et al.* designed a novel AMP that is specifically activated by the virulent proteases of *C. albicans* [161] (Figure 6). The AMP is composed of three domains, namely, an AMP (lactoferricin) as the active center, a protective peptide (magainin intervening sequence) that suppresses antimicrobial activity, and a specific linker that joins these two components and is efficiently cleaved by virulent proteases. The designed AMP is normally inactive because the anionic magainin intervening sequence neutralizes the positive charge of lactoferricin; the peptide is activated by cleavage of the specific linker by specific virulent proteases of *C. albicans*, and it subsequently kills *C. albicans*. The peptide demonstrated selective antimicrobial activity against *C. albicans*, but not against *Saccharomyces cerevisiae*, which does not possess any virulent protease. The AMP may also protect normal microflora, resulting in enhanced safety. Conjunction of additional selective domains that target other virulent attribute of *C. albicans* may further increase specificity and activity [162,163,164,165].

## 6. Conclusions

In recent years, the appearance of drug-resistant pathogens has provoked the need to develop novel antibiotics. Although AMPs have several promising properties, their clinical application has not been successful because of toxic side effects, rapid turnover, and low activity under physiological conditions. Several studies have dramatically improved the pharmacokinetics of AMPs *in vivo*; further improvements are still necessary.

Recently, designer AMPs have enabled temporal and spatial regulation of AMP activity; these designer AMPs possess ideal pharmacokinetic properties. Such drugs could increase the compliance of patients and improve their quality of life. Until now, few peptides have been used in the clinical setting so far, because of their unpredictable kinetics in mice and humans [166]. Therefore, further investigation with a focus on clinical trials to demonstrate effectiveness *in vivo* is required. Smart polymer technologies may improve the pharmacokinetics of designer AMPs. Smart polymers are now available to control the release of incorporated chemical compounds [167,168] and could also provide high stability against serum, proteolytic enzymes, and harsh acidic environments [169]. We believe that these efforts will produce innovative technologies and bear fruit that will benefit public health in the near future.
